# In Silico ADME and Toxicity Prediction of Benzimidazole Derivatives and Its Cobalt Coordination Compounds. Synthesis, Characterization and Crystal Structure

**DOI:** 10.3390/molecules27228011

**Published:** 2022-11-18

**Authors:** Anita Raducka, Marcin Świątkowski, Katarzyna Gobis, Paweł Szymański, Agnieszka Czylkowska

**Affiliations:** 1 Institute of General and Ecological Chemistry, Faculty of Chemistry, Lodz University of Technology, Żeromskiego 116, 90-924 Łódź, Poland; 2 Department of Organic Chemistry, Faculty of Pharmacy, Medical University of Gdansk, Gen. Hallera 107, 80-416 Gdańsk, Poland; 3 Department of Pharmaceutical Chemistry, Drug Analyses and Radiopharmacy, Faculty of Pharmacy, Medical University of Lodz, Muszyńskiego 1, 90-151 Lodz, Poland; 4 Department of Radiobiology and Radiation Protection, Military Institute of Hygiene and Epidemiology, 4 Kozielska St., 01-163 Warsaw, Poland

**Keywords:** transition metal complexes, coordination chemistry, crystal structure, benzimidazole derivatives, cancer chemotherapy, TG, FTIR, ADME analysis, bioinorganic medicinal chemistry

## Abstract

As a result of the synthesis, three new solids, cobalt (II) coordination compounds with benzimidazole derivatives, and chlorides were obtained. The ligands that were used in the synthesis were specially synthesized and were commercially unavailable. During the synthesis, a single crystal of the complex with the **L1** ligand was obtained and the crystal structure was refined. All coordination compounds were characterized by elemental analysis, infrared spectroscopy, and thermogravimetric analysis. All the obtained data allowed one to determine the formulas of the new compounds, as well as to determine the method of metal–ligand coordination. Thermal analysis allowed to know the temperature stability of the compounds, solids intermediate and final products of pyrolysis. Additionally, volatile decomposition and fragmentation products have been identified. The toxicity of the compounds and their bioavailability were determined using in silico methods. By predicting activity on cell lines, the potential use of compounds as chemotherapeutic agents has been specified. The blood-brain barrier crossing and the gastrointestinal absorption were defined. Pharmaceutical biodistribution was also simulated.

## 1. Introduction

Cobalt compounds are at the center of scientists’ interest, as evidenced by numerous literature reports. Cobalt is a transition element that has been present in medicine for years due to its applications [1,2,3,4,5]. As for the biological activity of this element, it has a wide range of applications. First of all, it is a trace element that is necessary for every living organism because it plays an important role in it. It occurs in the form of vitamin B12, otherwise known as cobalamin—a coordination compound in which cobalt is the central ion [6,7]. This vitamin is responsible for the regulation of erythrocyte production and is involved in the metabolism of nucleic acids and proteins. Vitamin B12 deficiency results in low cobalt concentrations and then causes blood clotting disorders [8]. In addition, an abnormal amount of this element in the body can cause many other disorders, including metabolic, neurological, and mental disorders [9,10,11]. Cobalt isotope ^60^Co is used in oncology in the form of a device emitting gamma radiation: the well-known cobalt bomb [12,13,14]. The drug of clinical significance, which was registered, was Doxovir (CTC-96), used in the treatment of labial herpes. Doxovir is a cobalt (III) compound with two axially coordinated 2-methylimidazole ligands [15,16]. From a coordination chemistry point of view, cobalt is a very interesting central ion, especially at a time when cancer, neurodegenerative diseases, and infections are a very serious public health problem. Therefore, the search for innovative and selective therapeutics is largely based on the design and synthesis of compounds capable of coordinating metal ions transitions involved in biological processes, especially when we plan the synthesis and select the appropriate ligands to design the drug for a specific disease. In this article, we focused on the preparation and characterization of cobalt coordination compounds with non-commercial benzimidazole derivatives. Our work is a continuation of previously published results with other metals. The ligands used in the synthesis have been specially designed for further conversion into coordination compounds. The choice fell on benzimidazole derivatives due to the wide spectrum of activity, and in particular, we took into account the antitumor properties of these compounds [17,18,19,20,21]. The above-mentioned activities, in combination with the properties of cobalt ions, led to the assumption that the newly formed compounds will show the expected cytostatic possibilities.

## 2. Results and Discussion

### 2.1. Synthesis

#### 2.1.1. Ligand Synthesis

**Scheme 1:**
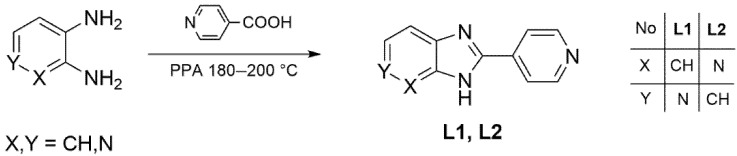
Synthesis of ligand **L1** and **L2**.

**Scheme 2:**
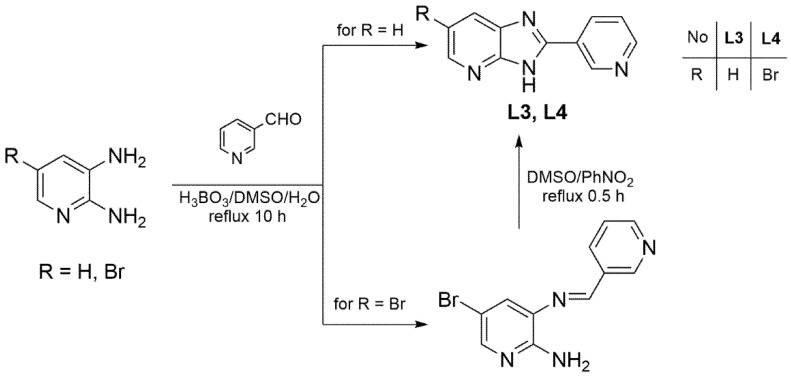
Synthesis of ligand **L3** and **L4**.

Ligands **L1** and **L2** were synthesized by the condensation and cyclization of 2,3-diminopyridine or 3,4-diaminopyridine with isonicotinic acid in PPA (polyphosphoric acid) (Scheme 1), and ligands **L3** and **L4** were obtained by reacting 2,3-diaminopyridine or 5-bromo-2,3-diaminopyridine with 3-pyridinecarboxaldehyde in the presence of boric acid in a mixture of water and DMSO (Scheme 2). We have previously described these syntheses [22,23,24].

#### 2.1.2. Complex Synthesis

**Scheme 3:**
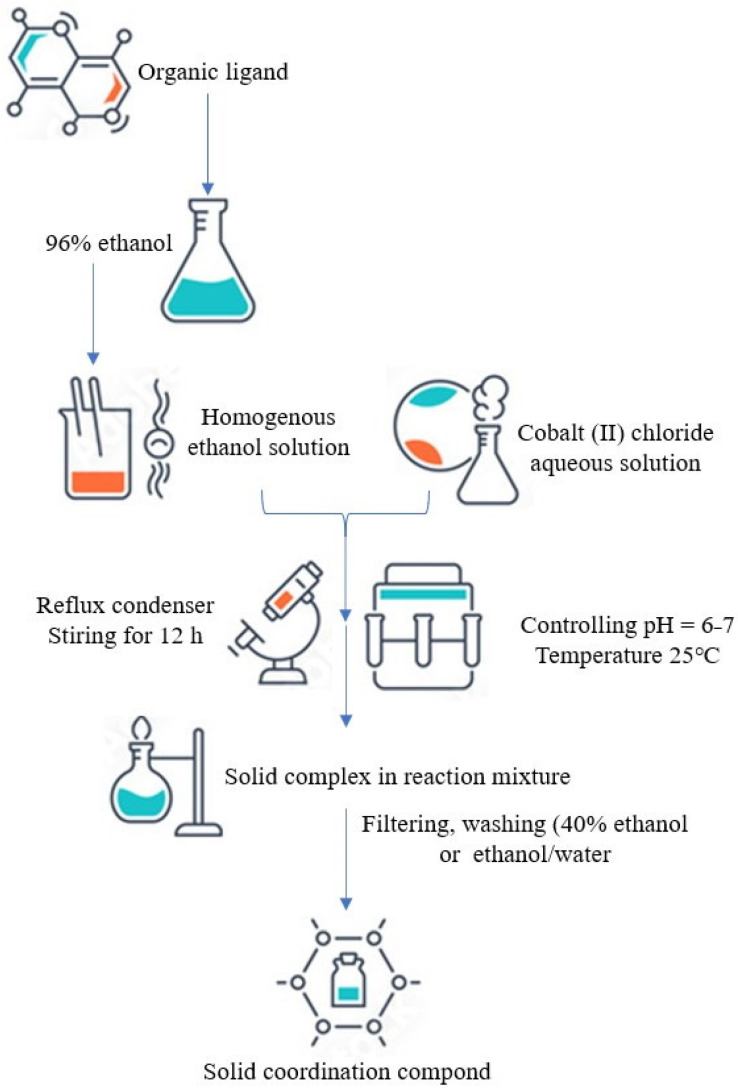
Synthesis of solid cobalt (II) coordination compounds.

All of the coordination compounds—**C1**, **C2**, and **C4**—were prepared according to the previously published scheme in articles [22,23,24] (Scheme 3). Unfortunately, despite numerous attempts to synthesize the complex **C3** with the **L3** ligand, this activity was not completed successfully. Nevertheless, the nomenclature of both the ligands and the compounds obtained has been retained. Benzimidazole derivatives (0.25 mmol) and cobalt chloride dihydrates (0.25 mmol) were dissolved at 96% *v*/*v* ethanol until homogeneous solutions were obtained. Using a reflux condenser and a magnetic stirrer, they were mixed for 12 h. The total volume of the reaction mixture was 30 mL. The reaction was carried out under constant room temperature (25 °C) and controlled pH (6–7) until the formation of a precipitate of coordination compounds, which was then washed with 40% EtOH and a mixture of EtOH and H_2_O (*v* = 1/1). The reaction products were air dried at room temperature. The obtained coordination compounds were defined by means of elemental C/H/N analysis and the determination of Co (II) content (Table 1), FTIR spectra, and the TG-MS technique, as well as by solving the crystalline structures of the obtained single crystals.

### 2.2. FTIR Spectra

**Figure 1:**
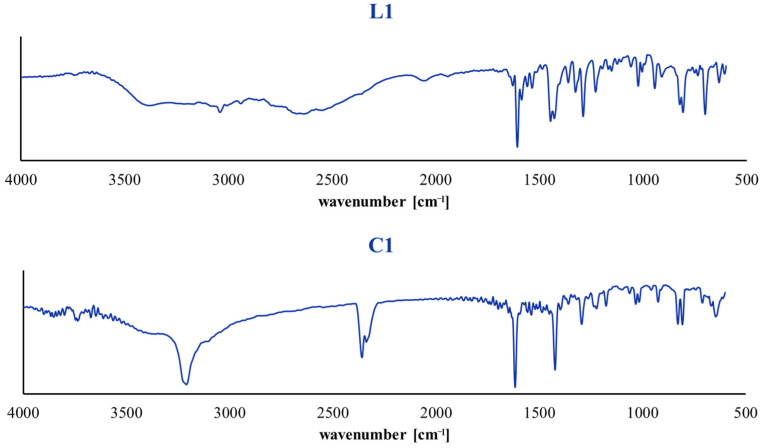
FTIR spectrum of free organic ligands **L1** and complex **C1**.

**Figure 2:**
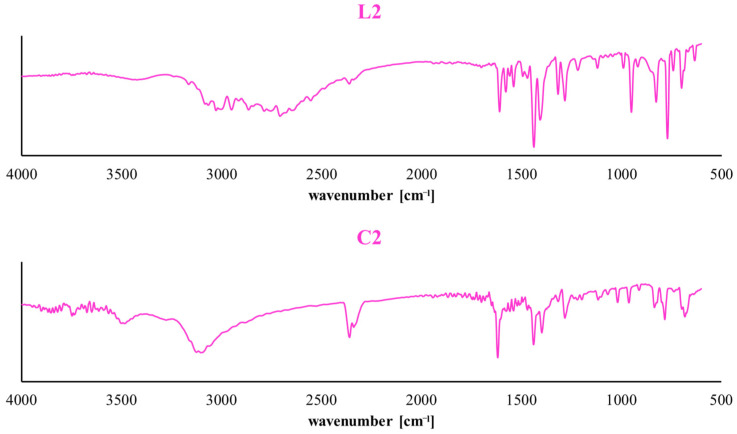
FTIR spectrum of free organic ligands **L2** and complex **C2**.

**Figure 3:**
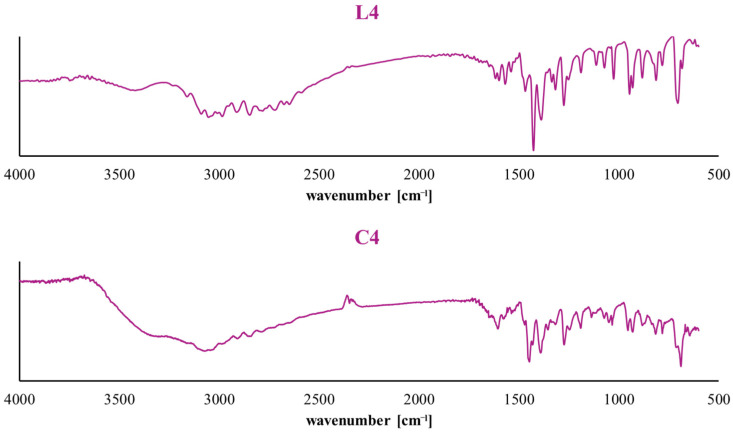
FTIR spectrum of free organic ligands **L4** and complex **C4**.

Figure 1, Figure 2 and Figure 3 show the pairwise spectra of the ligand and its corresponding cobalt complex compound. Infrared spectra are determined in the range 4000–500 cm ^−1^. In the infrared spectra in the case of the **C1** and **C4** compounds, we can observe a wide absorption band ν(OH) from water molecules in the range: 3450–3300 cm^−1^ for **C1** and 3600–3300 cm^−1^ for **C4**, We can also observe that the ligand bands that are preserved in the spectra of the complexes are shifted towards higher and lower wavenumbers. The free ligands show a characteristic band at 3500–2500 cm^−1^ that is associated with the ν(NH) and ν(CH) stretching modes. As expected, this band is present in the IR spectra of complexes but it is shifted into a higher wavelength in comparison to the free ligands. The vibration modes ν(C=N) and ν(C=C) in the free benzimidazole derivatives occur in the ranges 1609–1539 cm^−1^ and 1447–1411 cm^−1,^ respectively. For complex **C1,** they are at 1618, 1424 cm^−1^; for **C2**: 1618, 1439 cm^−1^; and in the case of **C4**: 1604, 1447 cm^−1^. All of these changes indicate coordination by a central ion.

### 2.3. Thermal Study

**Figure 4:**
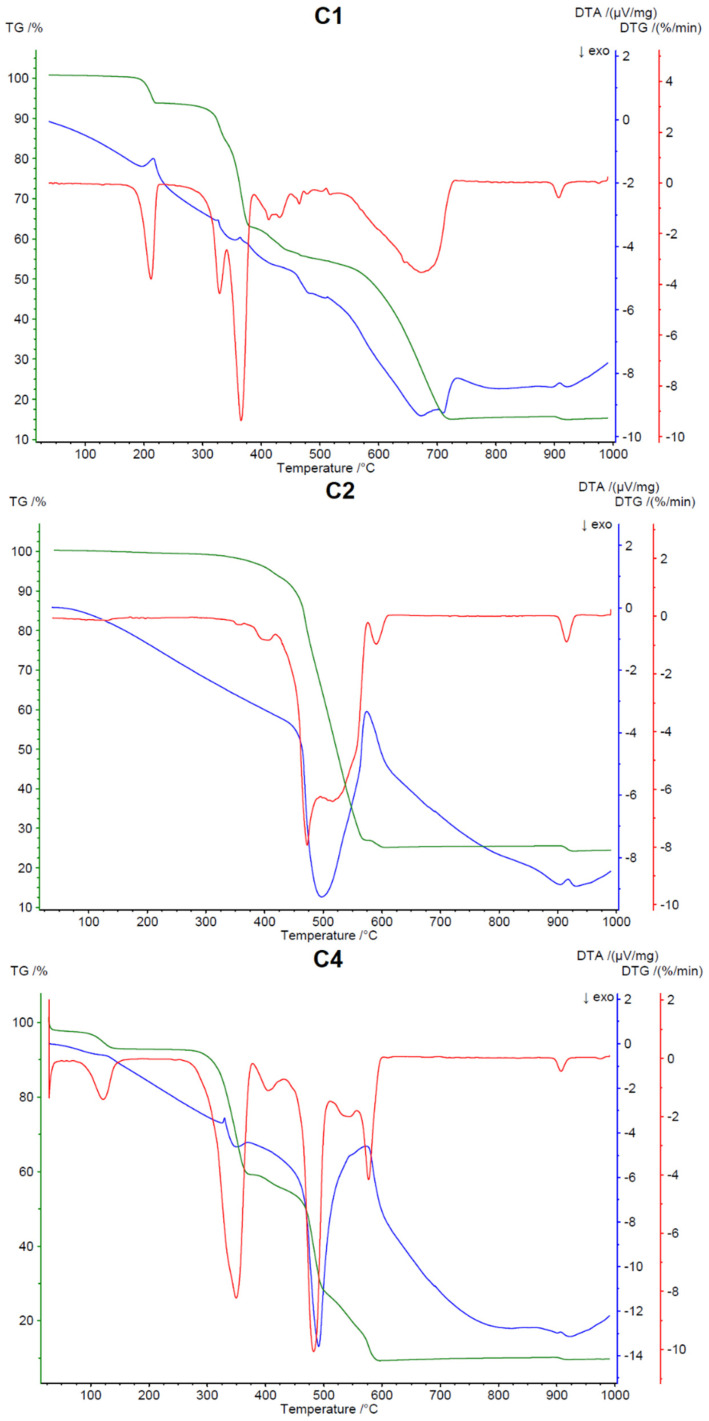
Thermal analyses of studied compounds **C1**, **C2,** and **C4**.

The thermal decompositions of **C1** and **C4** start from dehydration (Figure 4, Table 2). The high temperature of water removal for **C1** indicates that two water molecules are directly coordinated with the cobalt cation. In the case of **C4**, dehydration occurs in two stages. Their temperature ranges suggest that one water molecule is placed in the outer coordination sphere, while the other two are in the inner coordination sphere. The ligand decomposition begins at lower temperatures for **C1** and **C4** in comparison to anhydrous **C2**. However, these differences were not related to the presence of water for copper(II), zinc(II) and cadmium(II) coordination compounds containing the same ligands[22,23,24]. The most probable explanation for the greater temperature resistance of **C2** is a different compound structure. **C1** is a mononuclear compound (see Section 2.4), and based on previous structural findings [23], **C4** should also possess a discrete structure. The location of nitrogen atoms in **L2** suggests that **C2** can be a coordination polymer, and generally, polymeric compounds are more thermally stable than discrete compounds [25]. The ligand decomposition occurs in two stages [22,23,24], due to the fragmentation into the pyridine (which is removed first, *m/z* signals 26, 27, 50, and 52 [26]) and the imidazopyridine part. During the second stage, chlorides are removed simultaneously (*m/z* signals: 35, 36, 37, 70, and 72). The final solid product of decomposition is cobalt oxide in each case (confirmed by XRD study).

### 2.4. Structural Analysis

**Figure 5:**
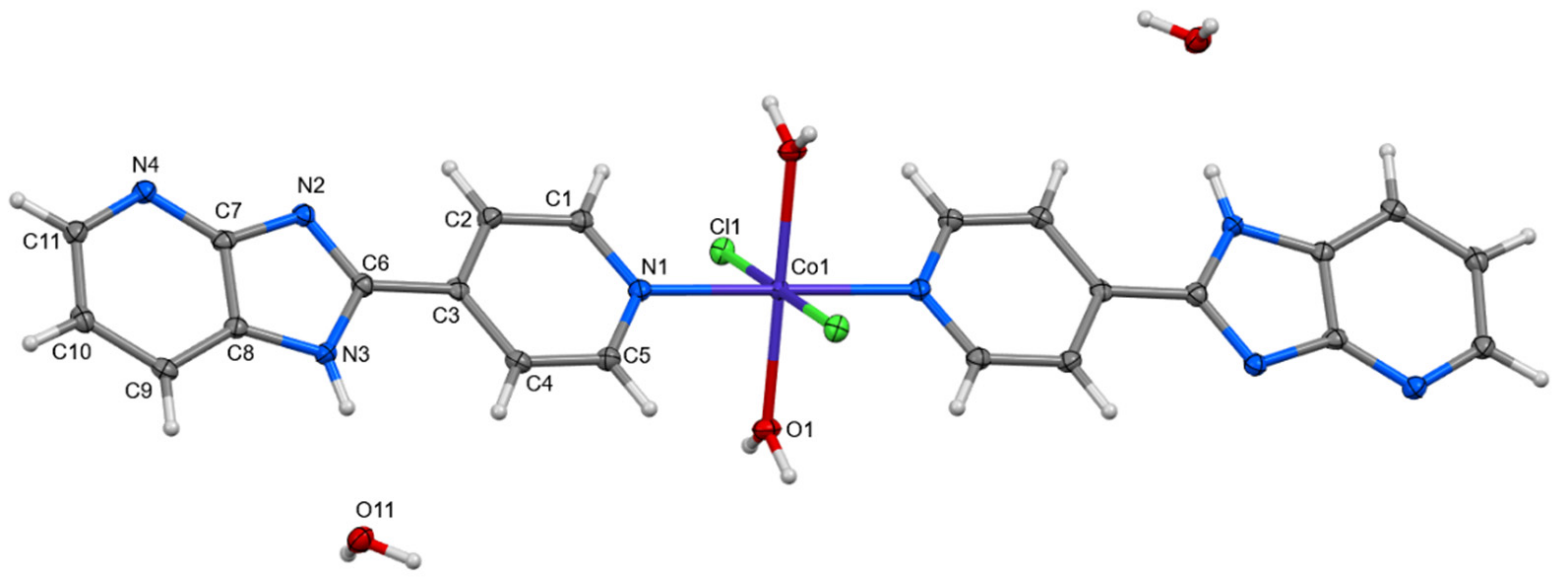
Molecular structure of **C1**.

**Figure 6:**
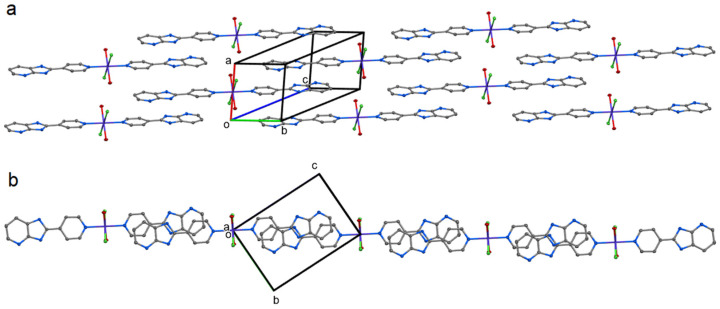
Molecular packing in **C1** showing π-stacking interactions: view on a π-stacking layer (**a**); view along a axis (**b**).

**Figure 7:**
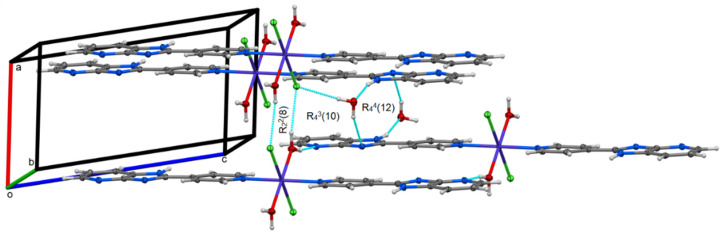
H-bond cyclic synthons in **C1**.

Crystals of **C1** left in the air for several seconds were becoming powder. Therefore, a suitable single crystal was placed on a diffractometer pin and mounted on a diffractometer in the nitrogen flow (100 K) immediately after removal from the synthesis solution. The X-ray measurement reveals that **C1** is a mononuclear coordination compound of the formula [CoCl_2_(L1)_2_(H_2_O)_2_]∙2H_2_O (Figure 5). According to elemental and thermal analyses, the formula of **C1** is [CoCl_2_(L1)_2_(H_2_O)_2_], which means that **C1** loses water molecules from the outer coordination sphere in the air and this is a consequence of the instability of its crystals. The cobalt cation is six-coordinated by two **L1** molecules (via a nitrogen of the pyridine ring), two chlorides, and two water molecules. The coordination polyhedron adopts almost perfect octahedral geometry (Table 3). The asymmetric unit contains half of the coordination entity due to the location of the central atom on the inversion center (special position *c* of the *P*-1 space group). **L1** molecules are almost planar. The dihedral angle between the plane of the pyridine ring and the plane of the imidazopyridine part is 3.0°. The supramolecular structure of **C1** is stabilized via π-stacking interactions occurring between **L1** molecules. They form head-to-tail synthons, according to which all of the L1 rings are involved in the associations (Table 4). The π-stacking interactions assemble coordination entities into layers along (0 1 −1) crystallographic planes (Figure 6). The stabilization between π-stacking layers is provided by OH···N, OH···Cl, and NH···O hydrogen bonds (H-bonds), which create the *C*(11)*C*(2)*D*(2)*D*(2)*D*(2) unitary graph set (Table 4). It is possible to distinguish three H-bond cyclic synthons, which can be described as the R_2_^2^(8), R_4_^3^(10), and R_4_^4^(12) graph motifs (Figure 7).

### 2.5. In Silico Methods

**Figure 8:**
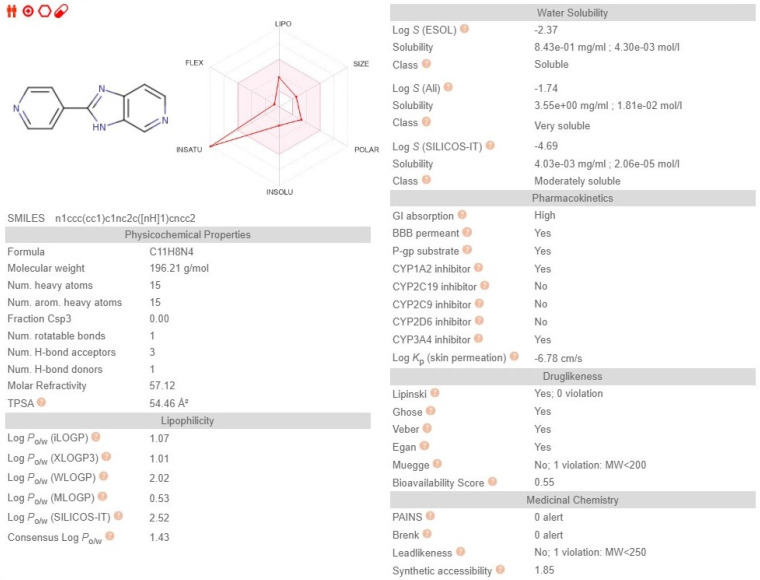
ADME for **L1**.

**Figure 9:**
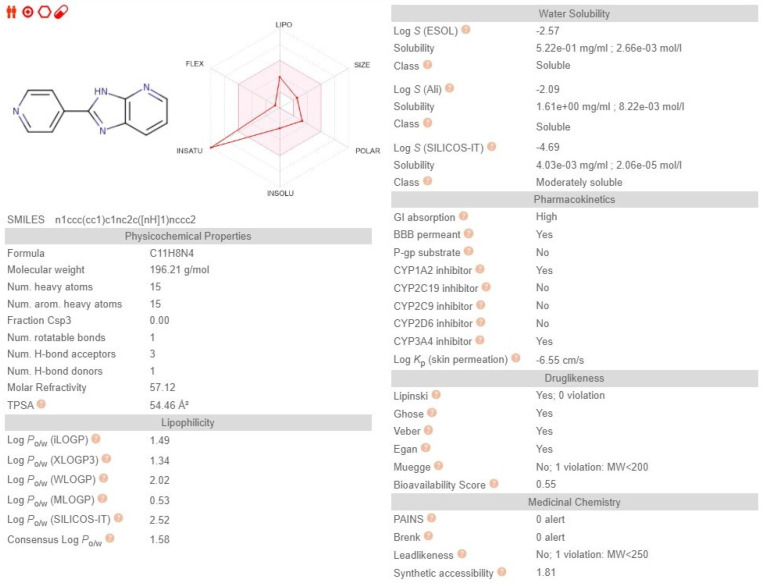
ADME for **L2**.

**Figure 10:**
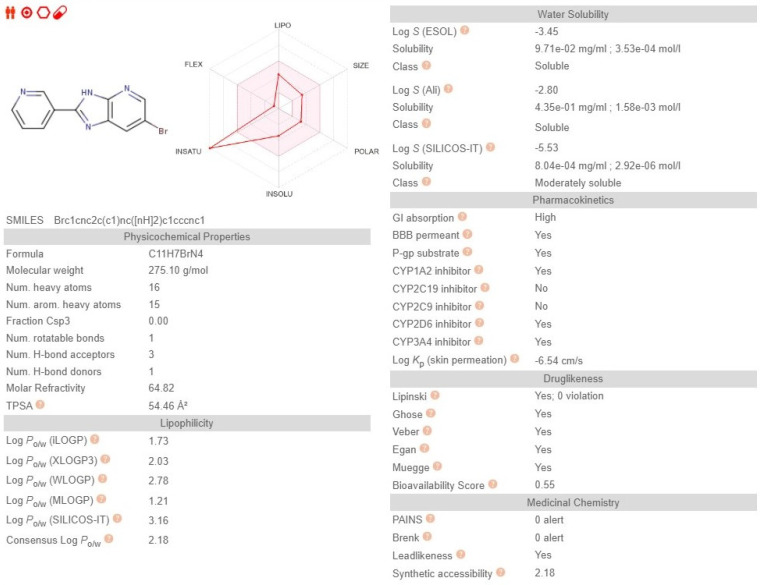
ADME for **L4**.

**Figure 11:**
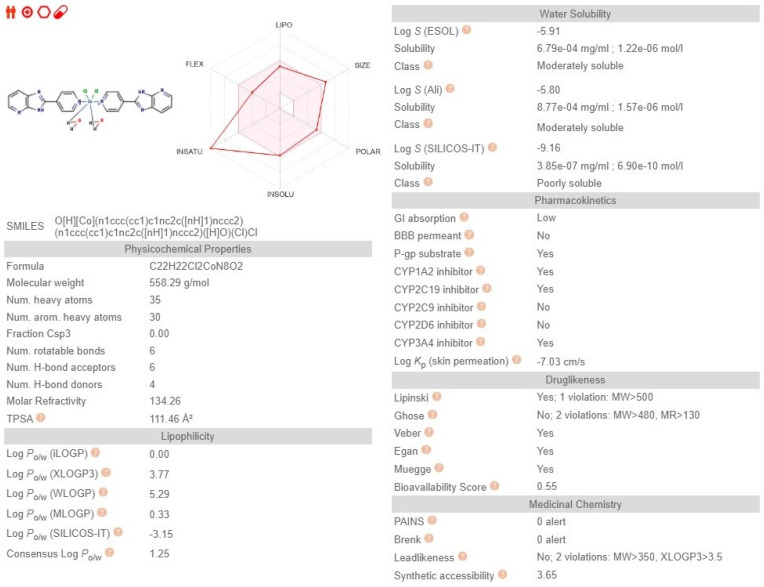
ADME for **C1**.

ADME analysis (absorption, distribution, metabolism, and excretion analysis) was performed for all free ligands and for the coordination compound for which a single crystalline was obtained. The bioavailability radars were made by the Swiss ADME service to assess the similarity of the molecule to the drug (Figure 8, Figure 9, Figure 10 and Figure 11).

**Figure 12:**
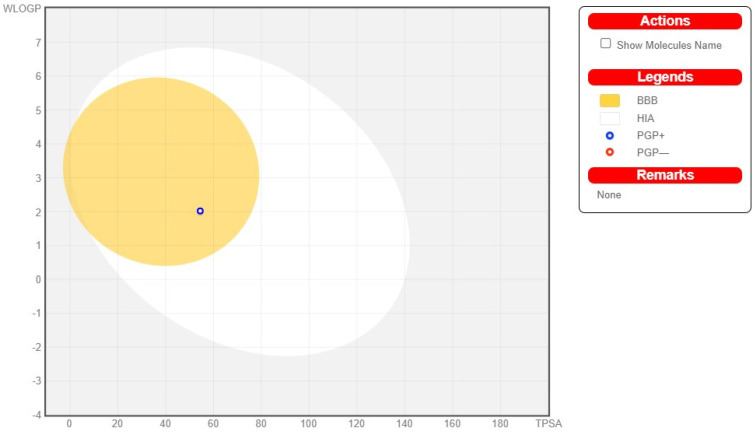
BOILED-Egg diagram for the ligand **L1**.

**Figure 13:**
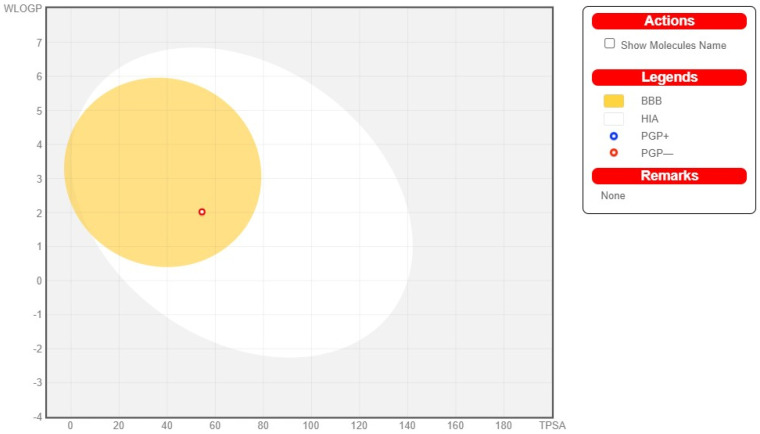
BOILED-Egg diagram for the ligand **L2**.

**Figure 14:**
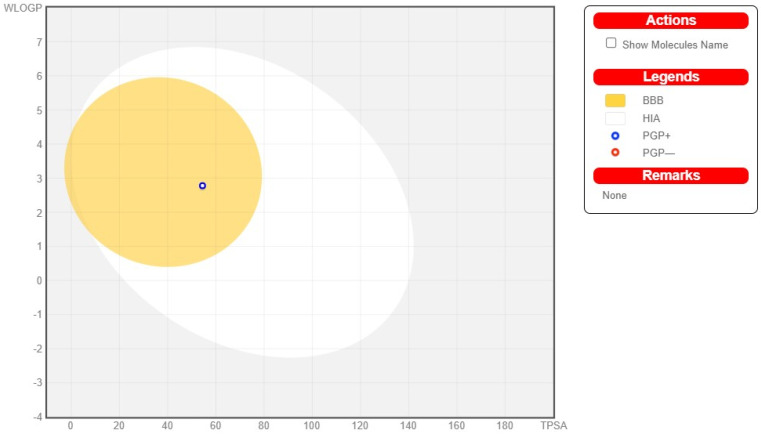
BOILED-Egg diagram for the ligand **L4**.

Lipophilicity (LIPO) for ligands was within the range 1.01 < XlogP3 < 2.03; for complex 3.77, polarity (POLAR) was within the range 54.56 Å2 < TPSA < 111.46 Å2, where TPSA (topological polar surface area). Ligands belong to the class of moderately soluble compounds, while the synthesized compound belongs to the class of poorly soluble compounds. Ligands **L1** and **L2** met the rules of Lipiński [27], Ghose [28], Veber [29], and Egan [30]; **L4** additionally satisfied the rule of Muegge [31], while the complex complied with all of the above-mentioned rules except the Ghose rule. All of the compounds are bioavailable on the level of 0.55. The BOILEDEgg graph (Brain Or IntestinaL EstimateD permeation method) was used to predict the gastrointestinal absorption and brain access of all ligands. These properties are crucial when searching for a new drug, and from this point of view all ligands are good candidates for being potential drugs (Figure 12, Figure 13 and Figure 14). Unfortunately, the complex for which the single crystal was obtained is an overly large molecule and therefore cannot be considered as a potential candidate.

**Figure 15:**
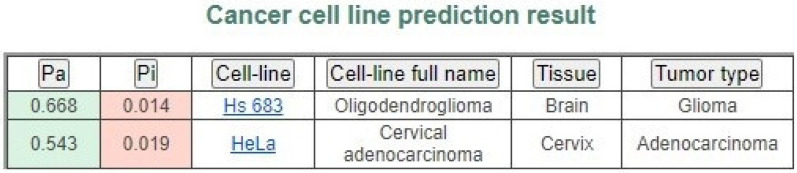
Cancer-cell-line prediction for **L1**.

**Figure 16:**
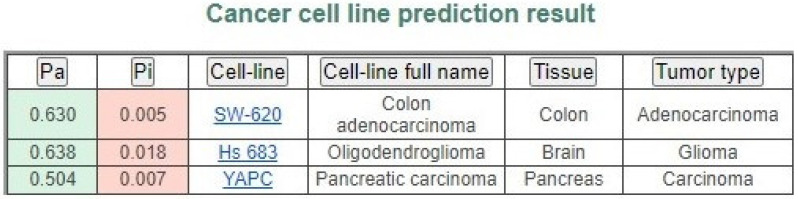
Cancer-cell-line prediction for **L2**.

**Figure 17:**
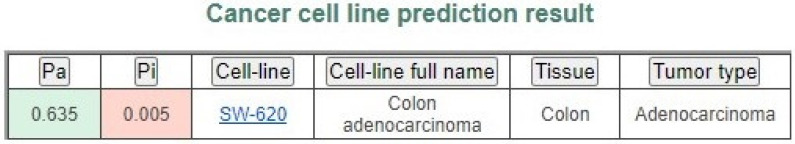
Cancer-cell-line prediction for **L4**.

The ligands have been classified by the Servis ProTox II into toxicity class 4 (predicted LD_50_: 1000 mg/kg). Predicting activity against cell lines using the Way2Drug service provided the following information (Figure 15, Figure 16 and Figure 17).

**Figure 18:**
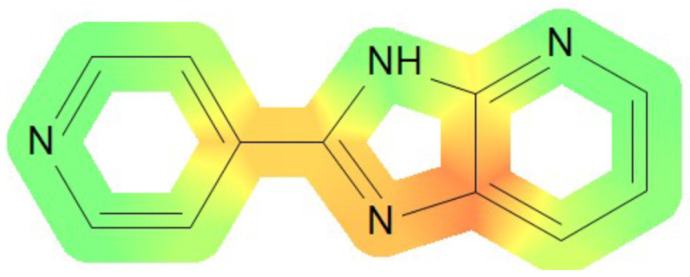
Ligand and in red genotoxic fragment.

The most useful predictions were made for the ligand and complex **C1**, which was the most interesting derivative among the series of compounds we studied. This calculation was made using ACD/Percepta software version 14.0.0 (Advanced Chemistry Development, Inc., Toronto, Canada). The binding to %PPB–91.6% proteins indicates a high probability of binding to plasma proteins, and this means that the ligand itself may persist in the blood for a long time so that its pharmacological effect may be prolonged. In contrast, in the case of a complex in which two ligand molecules are linked to a cobalt ion, the value drops to 32.89%. In the case of a coordination compound, the substance will reach the active site once at a higher concentration (this was confirmed by further simulations on pharmacokinetics). Analyzing the structure of the system for potential genotoxicity shows that it is limited and includes only a fragment (Figure 18) of the structure with a moderate potential to induce genotoxic effects on cells. A positive prediction among all of the positive Ames tests indicates the probability of a positive test 0.74 and increases in the case of the complex to 0.92. In the case of potential use of the substances as anticancer drugs, this may enhance their lethal effect.

**Figure 19:**
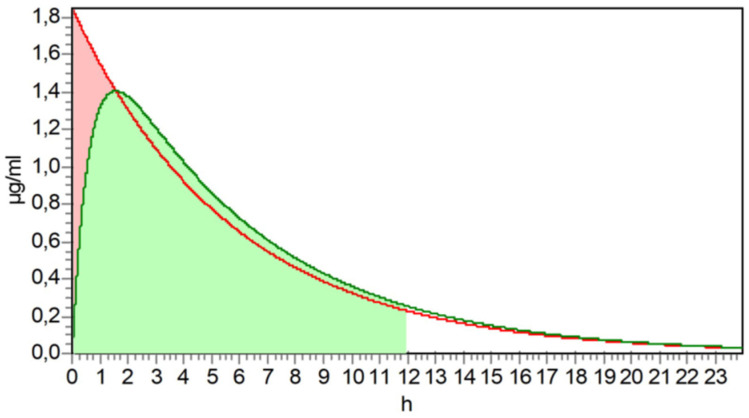

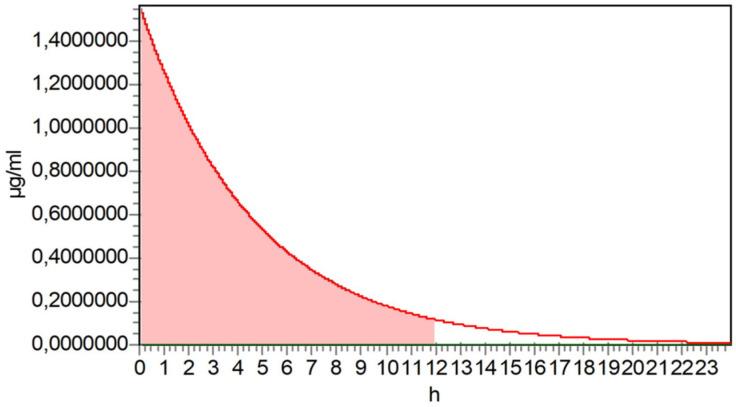
Pharmaceutical biodistribution for ligand **L1** and complex **C1**.

In terms of solubility, the analysis indicates that it would be appropriate to administer the substances via the per os (oral) route. Then, the molecules in the environment of the gastrointestinal tract have the greatest chance of protonation and increase solubility and absorption into the bloodstream. The substance that will be absorbed from the gastrointestinal tract will be mainly in the central compartment, i.e., circulating in the blood, as evidenced by the calculated predicted Vd-value of 0.35 for the ligand and 0.45 L/kg body weight for the complex. Thus, Vd is in the range of Vd −20 ÷ 40 L and tells us that the substance will reach about 60% of the sites in the human body where there is an aqueous environment. In terms of simulating and predicting pharmaceutical biodistribution by determining the area under the concentration-time curve (AUC), we can show that there are no significant differences between simulated doses of 50 mg and 500 mg. It seems interesting that in the case of the complex **C1**, the recommended route of administration is by injection. On the other hand, when predicting biodistribution over time for the ligand itself, the complex is comparable. The difference is that the simulation predicts the possibility of an oral route of administration only for the ligand. In summary, biodistribution expressed by potential concentration attainment over time appears to be comparable, and after about 12 h in both substances, the drug concentration is at a comparable level and may account for about 10% of the administered drug dose (Figure 19).

## 3. Materials and Methods

### 3.1. Materials and Analysis

The substrates used for ligand synthesis, as well as CoCl_2_·2H_2_O, were purchased from Sigma Aldrich (Warszawa, Poland).

### 3.2. Methods and Instruments

In order to compare the results of our work on coordination compounds with benzimidazole derivatives, the same techniques and methods were used. Samples of complexes (about 20 mg) were digested in a concentrated mixture of 36% HCl (1 mL) and 65% HNO_3_ (6 mL). The contents of Co (II) in the solid complexes were determined with an F-AAS spectrometer (Analytik Jena, contraAA 300, Jena, Germany) with a continuous source of light and using an air/acetylene flame (Analytik Jena, contraAA 300). Absorbances were measured at the analytical spectral lines at 242.5 nm for Co (II). The limit of quantification was 0.002 mg/L for Co (II). The solid samples were decomposed using an Anton Paar Multiwave 3000 (Graz, Austria) closed-system instrument. Mineralization was carried out for 45 min at 240 °C under a pressure of 60 bar. The contents of carbon, hydrogen, and nitrogen were determined with an instrument from Vario Micro Pharmaceutics 2022, 14, 1626 3 of 15 Company Elementar Analysensysteme GmbH (Langenselbold, Germany). FTIR spectra were recorded with an IR Tracer-100 Schimadzu Spectrometer (4000–600 cm^−1^ with an accuracy of recording of 1 cm^−1^, Kyoto, Japan) using KBr pellets. The thermal analyses, accompanied with analyses of volatile products, were carried out in the atmosphere of synthetic air (20% O_2_, 80% N_2_) on Netzsch STA 449 F1 Jupiter thermoanalyzer (Netzsch-Geratebau GmbH, Selb, Germany) coupled with Netzsch Aeolos Quadro QMS 403 mass spectrometer (Netzsch-Geratebau GmbH, Selb, Germany). The samples were heated within the temperature range of 30–1000 °C, with a heating rate of 10 °C/min. Mass spectra were registered every 15 s, within the *m/z* range of 10–200. X-ray diffraction data were collected for C1 on XtaLAB Synergy Dualflex Pilatus 300K diffractometer (Rigaku Corporation, Tokyo, Japan). Using Olex2 [32], the structures were solved with the SHELXT [33] using Intrinsic Phasing and refined with the SHELXL [34] using least-squares minimization. All non-hydrogen atoms were refined anisotropically. All hydrogen atoms were found from the Fourier difference map and refined using the “riding” model. The details concerning X-ray diffraction data and structure refinement are given in Table (Table 5).

### 3.3. In Silico Methods

An ADME analysis was performed using the SwissADME service (Swiss Institute of Bioinformatics 2021) [35,36,37] and the ProTOX II service for the prediction of the toxicities of the tested compounds [38]. Computer calculations were performed by ACD/Percepta version 14.0.0 (Advanced Chemistry Development, Inc., Toronto, ON, Canada).

## 4. Conclusions

The aim of the research was to design non-commercial ligands—derivatives of benzimidazoles—and to synthesize coordination compounds formed in the system: cobalt (II)–organic ligands–chlorides. As a result of the synthesis, four organic ligands were obtained, for which three new coordination compounds were synthesized: **C1**, **C2,** and **C4**. Unfortunately, due to the inability to dissolve **L3** ligand in most solvents, only the ligands **L1**, **L2,** and **L4** were coordinated. Additionally, a single crystal of the compound **C1** was measured. All compounds were characterized by elemental analysis, infrared spectral analysis, and thermal analysis. The thermal stability of the compounds was determined. The most thermally stable compound was **C2**. The intermediate and final solid products of thermolysis fully correlate with the signals obtained from mass spectrometry. ADME analysis was performed for the individual ligands and the **C1** complex. Ligands belong to the class of moderately soluble, while the **C1** complex belongs to the class of poorly soluble. Anticancer cell line prediction was also performed for the ligands, and the results highlight the potential for action against glioblastoma cells. For the **L1** ligand and the **C1** complex, binding to plasma proteins is predicted. The ligand can remain in the blood for a long time, and therefore its pharmacological effect can be prolonged. However, in the case of the complex in which two ligand molecules are attached to the cobalt ion, this value drops to 32.89%. In the case of a coordination compound, the substance reaches the active site in a higher concentration once.

## Figures and Tables

**Table 1:** Percentage composition of the elements in coordination compounds.

**C1** [Co(L1)_2_Cl_2_∙2H_2_O] (C_22_H_20_N_8_O_2_CoCl_2_) (558.2858 g/mol)	calc. Co 10.56 C 47.33 H 3.61 N 20.07 exp. Co 18.75 C 37.12 H 2.86 N 16.46
**C2** [Co(L2)_2_Cl_2_] (C_22_H_16_N_8_CoCl_2_) (522.2552 g/mol)	calc. Co 11.28 C 50.60 H 3.09 N 21.46 exp. Co 17.92 C 37.34 H 2.91 N 16.39
**C4** [Co(L4)_2_Cl_2_∙2H_2_O]∙H_2_O (C_22_H_20_N_8_O_3_CoBr_2_Cl_2_) (734.0932 g/mol)	calc. Co 8.03 C 35.99 H 2.75 N 15.26 exp. Co 14.62 C 30.69 H 2.15 N 13.54

**Table 2:** Temperature ranges, mass losses, and *m/z* signals of thermal-decomposition stages of studied compounds.

	C1	C2	C4	*m*/*z*
Dehydration	185–225 °C 6.3%	-	30–50 °C 2.2% 90–145 °C 4.2%	17, 18
1st stage of ligand decomposition	295–385 °C 30.0%	-	265–375 °C 33.4%	12, 17, 18, 22, 26, 27, 29, 30, 40, 44, 45, 46, 50, and 52
2nd stage of ligand decomposition and chlorides removal	385–725 °C 47.9%	375–600 °C 74.6%	375–595 °C 49.9%	12, 13, 17, 18, 26, 27, 29, 30, 35, 36, 37, 38, 41, 44, 45, 46, 70, 72, 79 *, 81 *, 158 *, 160 *, and 162 *
Final product (CoO)	15.8%	25.4%	10.3%	-

* signals present only in the mass spectra of **C4.**

**Table 3:** Structural data of the coordination polyhedron in **C1**.

i—j	d_ij_ (Å)	i—j—k	α_ijk_ (°)
Co1—Cl1	2.5086 (4)	Cl1—Co1—N1	90.72 (4)
Co1—N1	2.1419 (15)	Cl1—Zn1—O1	88.04 (4)
Co1—O1	2.0682 (12)	N1—Zn1—O1	89.90 (5)

**Table 4:** Hydrogen bonds and π-stacking interactions in the structures of C1. Each ring is indicated by one atom, which belongs solely to this ring. α is the dihedral angle between planes I and J, β is the angle between Cg(I)–Cg(J) vector and normal to plane I, and d_p_ is the perpendicular distance of Cg(I) on ring J plane.

D—H···A	d(D—H) (Å)	d(H···A) (Å)	d(D···A) (Å)	<(DHA) (°)
O1—H1A···N4 (i)	0.89	1.84	2.7226 (19)	170
O1—H1B···Cl1 (ii)	0.89	2.42	3.2626 (14)	159
N3—H3···O11	0.84	1.92	2.7458 (19)	164
O11—H11A···N2 (iii)	0.85	2.09	2.8966 (19)	158
O11—H11B···Cl1 (iv)	0.82	2.29	3.1083 (13)	172
R(I)···R(J)	d(Cg···Cg) (Å)	α (°)	β (°)	d_p_ (Å)
N1···N4 (v)	3.5335 (11)	0.03 (11)	23.4	3.2419 (8)
N1···N4 (vi)	3.7522 (11)	0.03 (11)	28.5	3.2988 (8)
N2···N2 (v)	3.6078 (11)	3.08 (10)	26.6	3.2946 (8)
N2···N2 (vi)	3.5451 (11)	3.08 (10)	20.5	3.2556 (8)

Symmetry transformations used to generate equivalent atoms: (i) *x*, *y*, *z* + 1; (ii) −*x* + 2, −*y* + 2, −*z +* 2; (iii) *x*, *y* − 1, *z*; (iv) −*x* + 2, −*y* + 1, −*z* + 1; (v) −*x* + 1, −*y* + 1, −*z +* 1; (vi) −*x* + 2, −*y* + 1, −*z +* 1.

**Table 5:** X-ray diffraction data, and structure-refinement details, for **C1**.

Empirical Formula	C_22_H_24_Cl_2_CoN_8_O_4_
Formula weight	594.32
Temperature (K)	100.0 (2)
Crystal system	triclinic
Space group	P-1
*a* (Å)	6.5580 (1)
*b* (Å)	8.3007 (1)
*c* (Å)	11.4458 (1)
*α* (°)	86.425 (1)
*β* (°)	81.127 (1)
*γ* (°)	74.308 (1)
Volume (Å^3^)	592.524 (13)
*Z*	1
Calculated density (g/cm^3^)	1.666
Absorption coefficient (mm^−1^)	8.181
*F(000)*	305.0
Crystal size (mm)	0.083 × 0.038 × 0.022
Radiation	Cu Kα (λ = 1.54184)
*θ* Range for data collection (°)	7.82 to 157.298
Index ranges	−7 ≤ h ≤ 8, −10 ≤ k ≤ 10, −14 ≤ l ≤ 13
Reflections collected	21,845
Independent reflections	2343 [R_int_ = 0.0580, R_sigma_ = 0.0239]
Data/restraints/parameters	2343/0/169
Goodness-of-fit on F^2^	1.161
Final R indexes [I ≥ 2σ (I)]	R_1_ = 0.0276, wR_2_ = 0.0756
Final R indexes (all data)	R_1_ = 0.0282, wR_2_ = 0.0759
Largest diff. peak/hole/e Å^−3^	0.32/−0.45

## Data Availability

Not applicable.

## Sample Availability

Samples of the compounds are not available from the authors.
